# Supplementary material for: Smooth Interpolating Curves with Local Control and Monotone Alternating Curvature
Source: Comput Graph Forum. 2022 Oct 6;41(5):25–38. doi: 10.1111/cgf.14600 (PMC9827861; doi:10.1111/cgf.14600)
Supplement: Supplementary file 1 — Supplement Material [file CGF-41-25-s001.zip › Local-Smooth-Interpolating-MonoCurvature/extern/clothoids/submodules/quarticRootsFlocke/docs/genindex.html]

Index — Quartic Roots v1.0.0

### Navigation

- index
- toc
- Quartic Roots »
- Index

# Index

**C**
| **I**
| **Q**
| **R**
| **V**

## C

|  |  |
| --- | --- |
| - complexType (C++ type) - Cubic (class) - Cubic::check (function) - Cubic::complexRoots (function) - Cubic::Cubic (function), [1] - Cubic::doubleRoot (function) - Cubic::eval (function), [1], [2] - Cubic::getNegativeRoots (function) - Cubic::getPositiveRoots (function) - Cubic::getRealRoots (function) - Cubic::getRoot0 (function), [1] - Cubic::getRoot1 (function), [1] | - Cubic::getRoot2 (function), [1] - Cubic::getRootsInOpenRange (function) - Cubic::getRootsInRange (function) - Cubic::info (function) - Cubic::numRoots (function) - Cubic::real\_root0 (function) - Cubic::real\_root1 (function) - Cubic::real\_root2 (function) - Cubic::root0 (function) - Cubic::root1 (function) - Cubic::root2 (function) - Cubic::setup (function) - Cubic::tripleRoot (function) |

## I

- indexType (C++ type)

## Q

|  |  |
| --- | --- |
| - Quadratic (class) - Quadratic::check (function) - Quadratic::complexRoots (function) - Quadratic::doubleRoot (function) - Quadratic::eval (function), [1], [2] - Quadratic::getNegativeRoots (function) - Quadratic::getPositiveRoots (function) - Quadratic::getRealRoots (function) - Quadratic::getRoot0 (function), [1] - Quadratic::getRoot1 (function), [1] - Quadratic::getRootsInOpenRange (function) - Quadratic::getRootsInRange (function) - Quadratic::info (function) - Quadratic::numRoots (function) - Quadratic::Quadratic (function), [1] - Quadratic::real\_root0 (function) - Quadratic::real\_root1 (function) - Quadratic::root0 (function) - Quadratic::root1 (function) - Quadratic::setup (function) - Quartic (class) - Quartic::check (function) - Quartic::eval (function), [1] | - Quartic::getNegativeRoots (function) - Quartic::getPositiveRoots (function) - Quartic::getRealRoots (function) - Quartic::getRoot0 (function), [1] - Quartic::getRoot1 (function), [1] - Quartic::getRoot2 (function), [1] - Quartic::getRoot3 (function), [1] - Quartic::getRootsInOpenRange (function) - Quartic::getRootsInRange (function) - Quartic::info (function) - Quartic::numComplexRoots (function) - Quartic::numRealRoots (function) - Quartic::numRoots (function) - Quartic::Quartic (function), [1] - Quartic::real\_root0 (function) - Quartic::real\_root1 (function) - Quartic::real\_root2 (function) - Quartic::real\_root3 (function) - Quartic::root0 (function) - Quartic::root1 (function) - Quartic::root2 (function) - Quartic::root3 (function) - Quartic::setup (function) |

## R

- roots (function)

## V

- valueType (C++ type)

### Quick search

### Table of Contents

- Quartic Roots Solver
- C++ API

«
hide menu

menu
sidebar
»

### Navigation

- index
- toc
- Quartic Roots »
- Index

© Copyright 2021, Enrico Bertolazzi.
Created using Sphinx 4.2.0.
